# Radiating pattern of surge-current-induced THz light in near-field and far-field zone

**DOI:** 10.1038/s41598-018-24673-9

**Published:** 2018-04-25

**Authors:** J. W. Han, Y. G. Choi, J. S. Lee

**Affiliations:** Department of Physics and Photon Science, School of Physics and Chemistry, Gwangju Institute of Science and Technology (GIST), Gwangju, 61005 South Korea

## Abstract

We generate the THz wave on the surface of an unbiased GaAs crystal by illuminating femtosecond laser pulses with a 45° incidence angle, and investigate its propagation properties comprehensively both in a near-field and in a far-field zone by performing a knife-edge scan measurement. In the near-field zone, i.e. 540 μm away from the generation point, we found that the beam simply takes a Gaussian shape of which width follows well a behavior predicted by a paraxial wave equation. In the far-field zone, on the other hand, it takes a highly anisotropic shape; whereas the beam profile maintains a Gaussian shape along the normal to the plane of incidence, it takes satellite peak structures along the direction in parallel to the plane of incidence. From the comparison with simulation results obtained by using a dipole radiation model, we demonstrated that this irregular beam pattern is attributed to the combined effect of the position-dependent phase retardation of the THz waves and the diffraction-limited size of the initial beam which lead to the interference of the waves in the far-field zone. Also, we found that this consideration accounting for a crossover of THz beam profile to the anisotropic non-Gaussian beam in the far-field zone can be applied for a comprehensive understanding of several other THz beam profiles obtained previously in different configurations.

## Introduction

Over the last few decades, an electromagnetic wave at the THz frequency has received a significant attention as it can provide a useful spectroscopic access to unveil the collective behaviors of electrons entangled with other degrees of freedom, i.e., orbital, spin, and lattice^1–7^. Quantitative information such as absorption coefficient or optical conductivity can be retrieved through a well-established THz time-domain spectroscopy technique which is often combined with far-field or near-field microscopy^8–11^. To precisely extract quantitative optical information, by the way, it is important to have an accurate knowledge regarding the beam profile at the interaction points of light with optical elements including the sample specimen of interest, and accordingly there have been extensive experimental and theoretical investigations about the THz beam pattern depending on its generation mechanism^12–14^. For the THz wave generated by the optical rectification in a nonlinear optical medium, e.g., ZnTe, Lin *et al*.^15,16^ demonstrated that a Gaussian aperture model successfully accounts for THz amplitude profile in both near-field and far-field regions. For the THz wave generated by a surge current in semiconductors such as GaAs and InAs, there have been several studies to characterize the beam profile, to our surprise, with no clear consensus made until now. Jepsen *et al*.^17^ reported that the THz pulse generated from the GaAs-based photo-conductive antenna has the Gaussian radiation pattern in the far-field zone. Reiten *et al*.^18^ performed the similar beam characterization and claimed that the THz beam consists of not only the simple Gaussian mode but also higher-order Laguerre-Gauss modes which later they attributed to a coupling effect of the THz pulse to a focusing optic^19^. Meanwhile, Jin *et al*.^20^ reported that the THz pulse emitted from the photo-Dember effect in InAs has the non-Gaussian amplitude distribution of which details are strongly frequency-dependent.

In this paper, we examine the beam profile evolution of the THz wave as it is generated from the un-biased free-standing semi-insulating (SI) GaAs, and propagates into the near-field zone and also to the far-field zone. We find that field patterns along the *y*-axis, i.e., normal to the plane of incidence, follow the Gaussian model in both the near-field and the far-field zone. On the other hands, along the *x*-axis, i.e., parallel to the plane of incidence, we find that the field pattern becomes strongly irregular as the beam propagates into the far-field zone. We discuss these anisotropic field distributions of the THz wave in terms of a numerical approach based on the dipole radiation model.

## Beam pattern at the generation point

**Figure 1:**
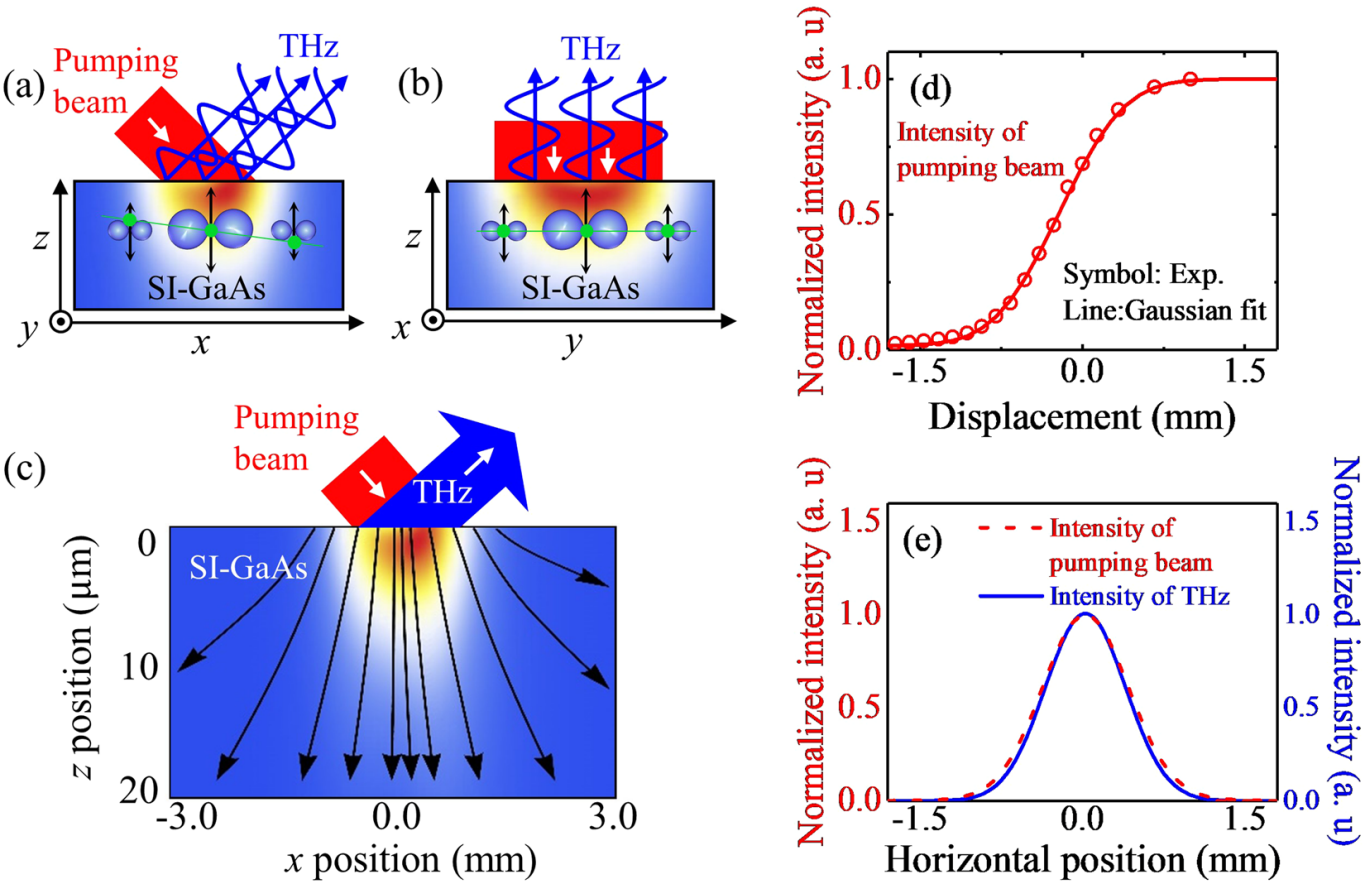
Schematic of the surge-current-induced THz emission from semi-insulating (SI) GaAs and characterization of the pumping beam and THz beam at the emitter surface. (**a**) The *xz* and (**b**) the *yz* cross-sections of the emitter with the pumping laser beam and the emitted THz wave indicated. Transient electric dipoles are located near the surface, and their magnitude and phase of the dipole moments are indicated by blue bubbles of different sizes and green lines, respectively. Whereas THz waves emitted from different positions of the emitter have the same phase at the surface, their phases at the wavefront become different in (**a**), but remain the same in (**b**). (**c**) Emitted THz field magnitude (false color) and the propagation direction (arrows), drawn in the *xz* plane of SI-GaAs. (**d**) Knife-edge scanning results of the pumping beam. (**e**) Normalized intensity profiles of the pumping beam (red dotted line) and the generated THz beam at the surface of SI-GaAs (blue solid line).

Before we discuss the THz beam profiles in the near- and far-field zone, let us first examine the radiation pattern of the THz wave at the generation point which will be used for further discussions about the wave propagation. As we do not have an experimental access to the beam characterization at the generation point, we provide only the computed intensity profile of the surge-current-induced THz wave upon its generation. To calculate the THz beam profile, the dipole radiation theory is employed, and local electric dipole moments are distributed at the surface to have specific position-dependences of their amplitude and phase^21,22^. The model begins from spreading 1000 electric dipole moments with an interval of 1.2 μm along the *x*-axis as shown in Fig. 1(a). Each electric dipole is induced by a drift motion of photocarriers due to the built-in potential, and hence its direction is uniformly defined to be surface normal. For the amplitude of the transient dipole moments, we assume a Gaussian distribution which simply follows an intensity distribution of the pumping beam. Meanwhile, by conducting the knife-edge experiment, we confirmed that the incident pumping beam is fully collimated and takes a simple Gaussian form with a full width at half maximum of 1.2 mm (line in Fig. 1(d)). Since the incidence angle of the pumping beam is 45° to the surface normal, the creation time of each point dipole should have a linear retardation along the *x*-direction. Here, we assume that the THz generation by a transient dipole and the THz propagation occur with the same speed, and the position-dependent phase variations are determined mainly by a simple geometrical factor, i.e., incidence angle of the pumping beam. Therefore, we consider a linear variation of the oscillation phase of the dipole moment with a difference by 4 ps for two dipoles located at each end (see a yellow diamond in Fig. 1(a)). Given with such dipole moment arrangement having specific amplitude and phase distributions, we simply applied a dipole radiation theory to obtain the THz electric field emitted from each local dipole moment, and summate all the contribution to determine the beam profile at the given detection point.

Figure 1(c) displays a false color image of a cross-sectional distribution of the THz electric field magnitude at 0.5 THz at the inside of the emitter. As we have considered the complex refractive index *n*_E_ (3.58 + i0.02 in a THz region) of SI-GaAs, a refraction of the beam can be clearly recognized from the color distribution. In particular, when we plot a line profile of the field magnitude along the *x*-direction (Fig. 1(e)), we can confirm that the THz beam at the generation point has an ideal Gaussian profile of which distribution follows the intensity profile of the input pumping beam.

## Beam pattern in the near-field zone

In the actual experiment, THz wave is generated by illuminating a femtosecond laser pulse on to the un-biased freestanding GaAs (001) crystal with an incidence angle of 45 degrees. The pumping beam has a wavelength 800 nm, and its photon energy 1.55 eV is slightly larger than the band gap energy of SI-GaAs. The pumping power is 160 mW, and the fluence is 0.25 J/cm^2^. To collect the THz wave in a reflection geometry, we use a pair of parabolic mirrors of which diameter and focal length are two and four inches, respectively. And, a photo-conductive antenna (BATOP corp.) is employed as the THz light detector.

**Figure 2:**
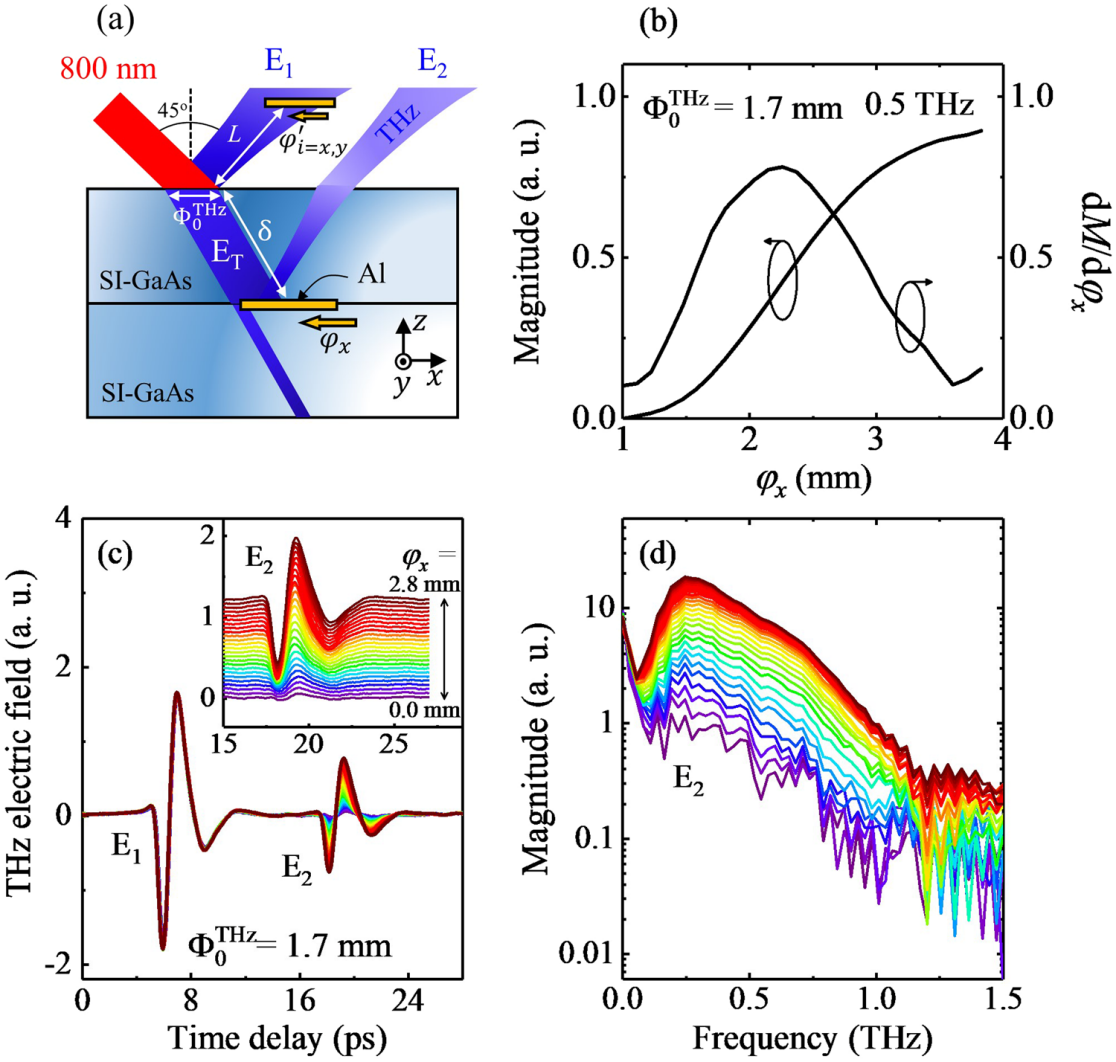
(**a**) Schematic diagram of the knife-edge experiment. Pumping beam of an 800 nm laser illuminates the surface of SI-GaAs and it is converted to the THz pulse E_1_, transmitted pulse E_T_, and reflected pulse E_2_ from the back side of the emitter. Traveling length of the THz wave in the emitter is indicated by δ. *φ* indicates the displacement of the knife-edge scanning and its direction is denoted by using subscriptions, i.e., *x* and *y*. (**b**) Magnitude profile at 0.5 THz. (**c**) Time-dependent profiles of THz electric field obtained with a variation of *φ*_*x*_. The inset shows a magnified view of E_2_ where each curve is shifted upward for the clarity. (**d**) Magnitude spectra corresponding to E_2_ where the same color profiles are used as in (**c**).

Let us then examine the THz field pattern in the near-field zone. Since the near-field zone is still too close to put a conventional knife edge, we here introduce an alternative approach. Figure 2(a) presents a sketch of the experimental geometry. To make a knife edge for the characterization of the THz beam at the backside of the emitter, we evaporate an 80 nm thick Al layer with a sharp edge on the half of the entire surface area of SI-GaAs and attach it to the backside of the emitter by gently applying a mechanical force (see inset of Fig. 2(a)). Note that the SI-GaAs with the Al knife on it is of the same kind with that used for the THz emitter which both have a dimension of 10 × 10 × 0.53 mm^3^. THz wave is generated at the top surface of the emitter and propagates into its inside. By sliding this assembly of the homogenous emitter and the knife edge, we can mimic the conventional knife-edge experiment^23,24^; the THz wave is largely reflected from the coated region, and it is detected in a reflection geometry. From this, we can investigate the beam profile at the back side of the emitter which is 540 μm (δ) away from the generation point.

Figure 2(c) displays the time-domain profiles of THz electric field obtained by displacing the knife edge. Two distinct wave packets are detected; the former one (E_1_) is the THz wave generated backwardly at the emitter surface, and the latter one (E_2_) is the THz wave generated forwardly and reflected backwardly at the backside of the emitter. Hence the temporal separation of about 12 ps exactly corresponds to the traveling time of the THz wave inside of the medium with an effective thickness of 540 μm. With a variation of the position of the knife edge *φ*_*x*_, whereas E_1_ remains the same, as expected, E_2_ exhibits dramatic changes; whereas E_2_ has an almost featureless waveform at *φ*_*x*_ = 0 mm, E_2_ increases its magnitude with an increase of *φ*_*x*_, and it reaches to have a maximal magnitude of about one-third of E_1_ at *φ*_*x*_ = 2.8 mm. Actually, E_2_ at *φ*_*x*_ = 2.8 mm can be well reproduced by considering a full reflection of the THz wave at the emitter-Al interface. And, a small electric field at *φ*_*x*_ = 0 mm may originate from the tiny air-gap formed between two SI-GaAs layers.

Figure 2(d) displays THz magnitude spectra of E_2_ with a variation of *φ*_*x*_. We pick up the magnitude at 0.5 THz from E_2_ spectra and display its *φ*_*x*_-dependence in Fig. 2(b). It shows a typical behavior of one-dimensional knife-edge scan result, indicating that our proposed experimental geometry seems to be possible to successfully assess the near-field zone beam profile. In addition, we also display a derivative of the amplitude profile, i.e., d*M*/d*φ*_*x*_. Although it does not fully reflect the original beam shape, it helps us to get a rough idea about the beam shape without any model fitting; d*M*/d*φ*_*x*_ shows the Gaussian-like profile, and its width seems to have about 1.8 mm, in accordance with the length scale of the THz beam waist at the generation point, i.e., ${{\rm{\Phi }}}_{0}^{{\rm{THz}}}$.

**Figure 3:**
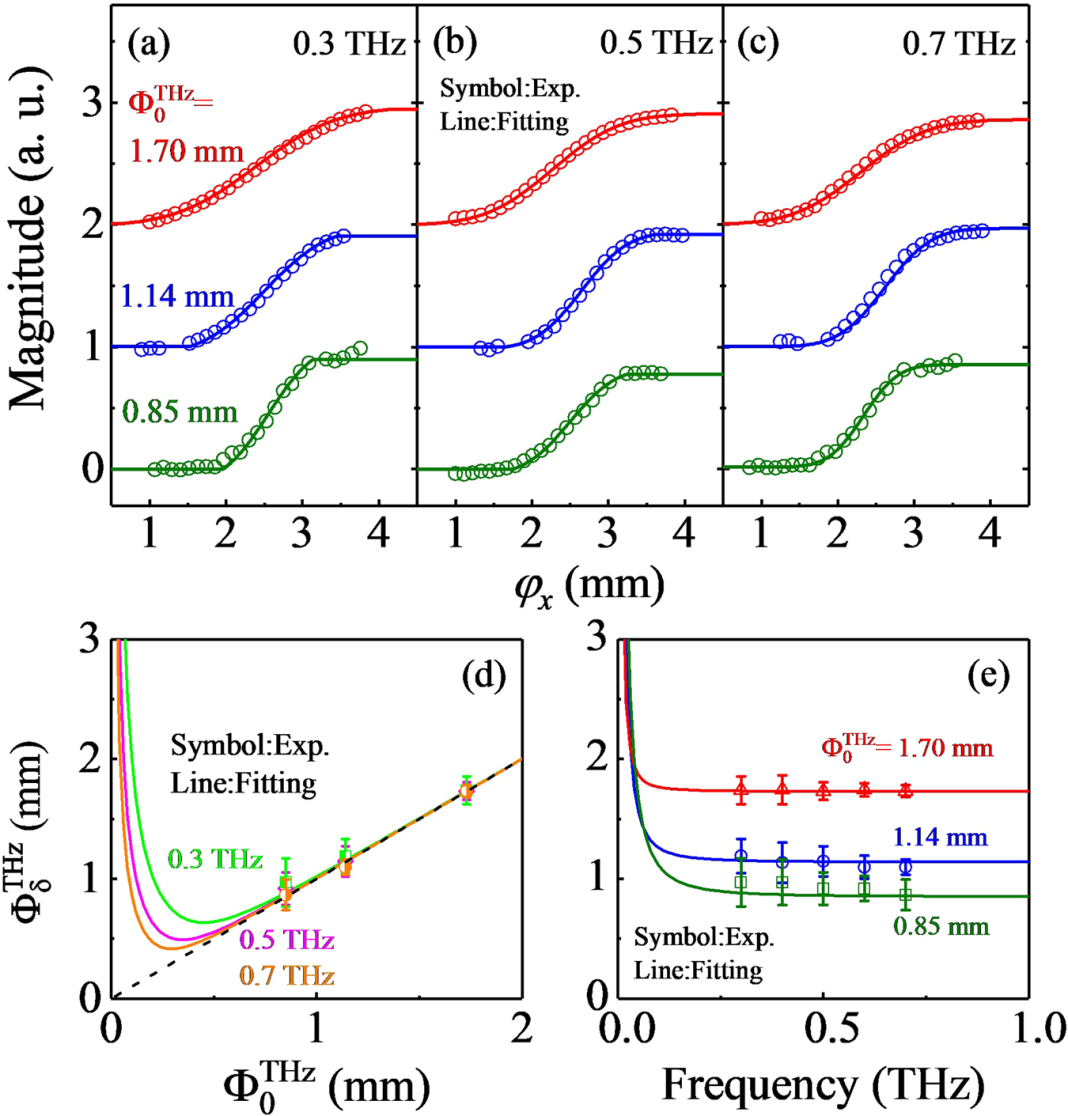
Results of the knife-edge experiment in the near-field zone with varying the initial beam waist ${{\rm{\Phi }}}_{0}^{{\rm{THz}}}\,\,$and the frequency: (**a**) 0.3 THz, (**b**) 0.5 THz, and (**c**) 0.7 THz. Estimated THz beam waist ${{\rm{\Phi }}}_{\delta }^{{\rm{T}}{\rm{H}}{\rm{z}}}$ as a function of ${{\rm{\Phi }}}_{0}^{{\rm{THz}}}$ (**d**) and the frequency (**e**). Symbols denote experiment results and the solid lines show the Gaussian model fit. The dotted line in (**d**) is a guideline to the eye indicating ${\,{\rm{\Phi }}}_{{\rm{\delta }}}^{{\rm{THz}}}={{\rm{\Phi }}}_{0}^{{\rm{THz}}}$.

From these knife-edge experimental results obtained in the near-field zone, we examine THz beam profiles in more detail particularly with a variation of the frequency. Figure 3(a–c) show magnitude profiles of the THz wave at 0.3, 0.5, and 0.7 THz. In each plot, there are three sets of the result which are obtained by varying the initial size of the THz beam as ${{\rm{\Phi }}}_{0}^{{\rm{THz}}}$ = 0.85, 1.14, and 1.70 mm. For all the cases, the detected magnitude of the THz wave varies monotonically with a variation of the knife edge position *φ*_*x*_. And, this is a typical feature of the simple Gaussian beam as demonstrated in Fig. 1. At the given frequency, the increase in ${{\rm{\Phi }}}_{0}^{{\rm{THz}}}$ from 0.85 to 1.70 mm leads to a visible difference in the results, i.e., a sharper increase before and after the saturation levels. At the given ${{\rm{\Phi }}}_{0}^{{\rm{THz}}}$, on the other hand, the change in the frequency from 0.3 to 0.7 THz seems to have little influence on the results.

We fit these results by adopting a Gaussian beam profile and display the full-width at half maximum as a function of the initial beam size and the light frequency in Fig. 3(d and e), respectively. To simulate the observed knife-edge results, we carry out the two-dimensional integration for the two-dimensional Gaussian beam as

1 $$M({\phi }_{x})={\int }_{-\infty }^{\infty }{\int }_{-\infty }^{{\phi }_{x}}{M}_{0}\exp \,[-\frac{{(x-{x}_{0})}^{2}}{2\times {({{\rm{\Phi }}}_{{\rm{\delta }}}^{{\rm{THz}}})}^{2}}-\frac{{(y-{y}_{0})}^{2}}{\frac{2}{\sqrt{2}}\times {({{\rm{\Phi }}}_{{\rm{\delta }}}^{{\rm{THz}}})}^{2}}]\,dxdy.\,$$

Here, *M*_0_ is the maximum magnitude at (*x*_0_, *y*_0_). To treat the anisotropic shape of the THz beam arising from a 45° incidence angle, we set the ${{\rm{\Phi }}}_{{\rm{\delta }}}^{{\rm{THz}}}$ along the *x*-axis is larger than that along the *y*-axis by a factor of $\sqrt{2}$. Whereas a pair of parabolic mirrors are used to guide the THz wave to the detector, the THz beam may be only partially collected when the beam size is large enough to overfill the first mirror. This effect can occur more easily when ${{\rm{\Phi }}}_{0}^{{\rm{THz}}}$ and the frequency decrease, and it actually appears as sharp truncations near the saturation points, for example, at ${{\rm{\Phi }}}_{0}^{{\rm{THz}}}=\,$0.85 mm and 0.3 THz (Fig. 3(a)). To account for this effect, we propagate the THz wave to the position of the first parabolic mirror by considering the paraxial wave equation for the Gaussian beam and collect the THz wave only within its area. The solid lines in Fig. 3(a–c) indicate the best fitting curves and the parameters used are displayed in Fig. 3(d and e). A decrease in the initial beam size is surely reducing the beam size in the near-field zone, i.e., at 570 μm away from the source point. On the other hand, we found no meaningful variation in the beam size when the light frequency is changed between 0.3 and 0.7 THz.

These tendencies can be described well by the Gaussian beam model derived from the paraxial wave equation. According to this, the beam waist ${{\rm{\Phi }}}_{{\rm{\delta }}}^{{\rm{THz}}}$ at the position δ is given as

2 $${{\rm{\Phi }}}_{{\rm{\delta }}}^{{\rm{T}}{\rm{H}}{\rm{z}}}={{\rm{\Phi }}}_{0}^{{\rm{T}}{\rm{H}}{\rm{z}}}\sqrt{1+{({\delta \xi }^{2}/{\delta }_{{\rm{R}}})}^{2}}.$$

Here, δ_R_ = π*n*(${{\rm{\Phi }}}_{0}^{{\rm{THz}}}$)^2^/4λ is a Rayleigh length where *n* is the complex refractive index of the medium and λ is the wavelength of light in the vacuum. A quality factor ξ^2^ is included to account for a deviation of the beam from the ideal Gaussian beam^25^. Solid lines in Fig. 3(d and e) are predictions based on Eq. (2) with ξ^2^ = 1, and explain the experimental results well^26^. Furthermore, the beam waist is almost unchanged from the initial one; a dotted line in Fig. 3(d) corresponds to ${{\rm{\Phi }}}_{{\rm{\delta }}}^{{\rm{THz}}}={{\rm{\Phi }}}_{0}^{{\rm{THz}}}$, and all the data points fall on this curve. This suggests that the measured point at δ = 570 µm is within the Rayleigh length. Together with the fitting results in Fig. 3(a and c), these comparisons confirm that the THz beam in the near-field zone is described by a simple Gaussian beam.

## Beam pattern in the far-field zone

**Figure 4:**
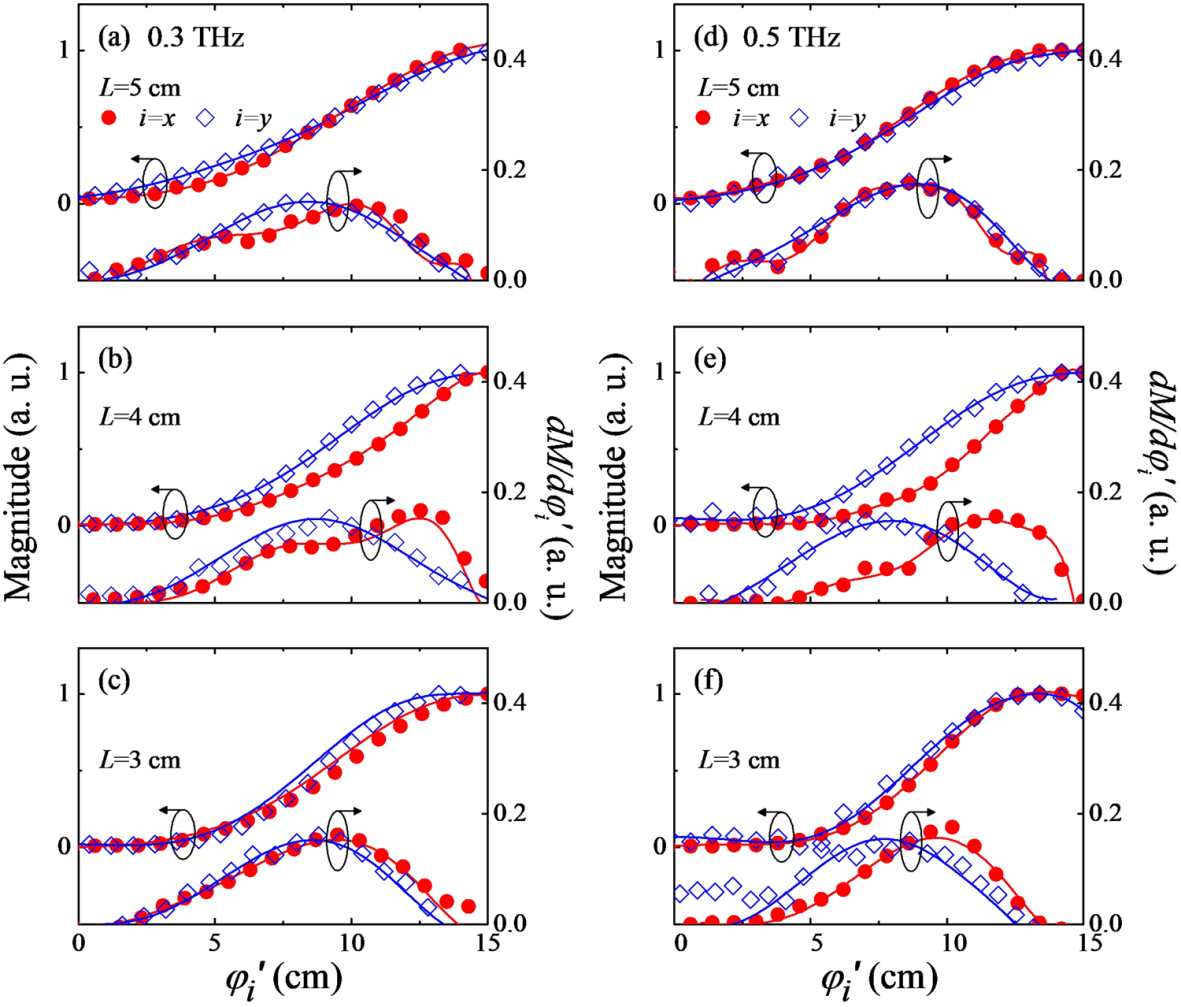
Dual axis knife-edge experiment results (*x*- and *y*-axis) obtained at two different frequencies of 0.3 THz (**a**–**c**) and 0.5 THz (**d**–**f**) and three different positions of *L* = 3, 4, and 5 cm. The red closed circles and the blue open diamonds denote the results of the *x* scanning and *y* scanning, respectively. All lines are obtained from the polynomial fit and inserted for the clarity.

We now discuss how the THz wave evolves further during its propagation in the far-field zone. In this far-field characterization, we locate a conventional knife-edge at the distance *L* apart from the generation point (Fig. 2(a)), and vary its position (*φ*_*i*=*x*,*y*_) along both *x*- and *y*-directions to obtain the two-dimensional profile of the beam intensity. Figure 4 displays the two-dimensional knife-edge scan results obtained at three positions of *L* = 3, 4, and 5 cm. In each case, the magnitude and the corresponding derivation profiles d*M*/d*φ*_*i*=*x*,*y*_ are shown at two frequencies, i.e., at 0.3 and 0.5 THz. For a guide to the eye, we also present solid lines following data points which are obtained by a simple polynomial fit. At *L* = 3 cm, the magnitude changes smoothly with seemingly no discernible anomalies along both *x*- and *y*-directions. Accordingly, a global maximum appears in deviation profiles d*M*/d*φ*_*i*=*x*,*y*_. Actually, such features evoke the Gaussian profile that we confirmed in the near-field zone. At *L* = 4 cm, significant changes are observed particularly from d*M*/d*φ*_*i*=*x*,*y*_; whereas the scan results along the *y*-axis look similar with the results at *L*=3 cm, d*M*/d*φ*_*i*=*x*_ exhibits a highly off-centered global maximum with double local maxima (Fig. 4(b and e)). Upon a further propagation of the wave to *L* = 5 cm, such asymmetric profile of the derivative cure becomes less pronounced, and it looks rather similar to the results at *L* = 3 cm. These results can be summarized as (i) the THz beam profile is given anisotropically in the far-field zone, e.g., at *L* ≥ 4 cm, (ii) its distribution along the *x*-direction strongly deviates from the simple Gaussian model, and (iii) details of the beam profile exhibit a large variation as the beam propagates. These observations at *L* ≥ 4 cm cannot be explained by the Gaussian beam propagation theory, and suggest that some intriguing physical effects come into play when THz beam propagates from the near-field to the far-field zone.

**Figure 5:**
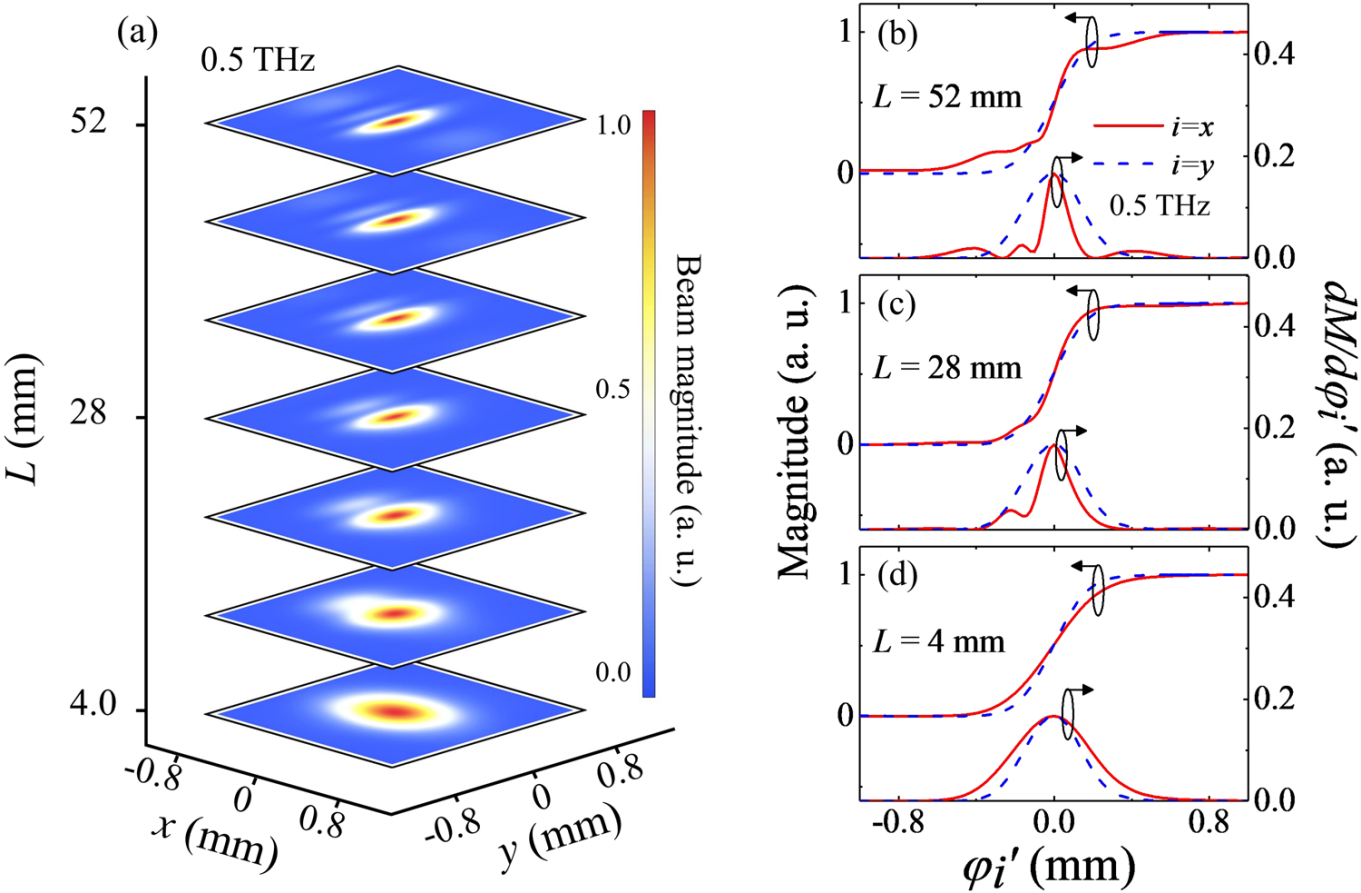
(**a**) Two-dimensional THz beam magnitude profiles computed at 0.5 THz with an increase of the propagation length *L* from 4 mm to 52 mm. (**b**–**d**) Knife-edge magnitude profiles (left scale) and corresponding differential profiles (right scale) computed from the magnitude profiles shown in (**a**). The results along *x*- (red line) and *y*-axis (blue line) are shown at three different positions of *L* = 4, 28, and 52 mm.

To get an insight into the obtained results, we compute two-dimensional beam profiles in the far-field regime based on the electric dipole model as done for the generation point. Note that the basic details of the simulation are the same with those for the beam profile at the generation point demonstrated in the section 2. By the way, we obtain one-dimensional profiles along *x*- and *y*-axes individually, and then multiply them to retrieve the two-dimensional magnitude profile. Figure 5(a) displays how the two-dimensional distribution of THz amplitude evolves out as the THz wave travels in the far-field region of 4 ≤ *L* ≤ 52 mm. Here, the frequency is set to 0.5 THz. To focus on the amplitude distribution, we normalize beam widths with respect to the width of the ideal Gaussian beams at the corresponding propagation distances. At *L* = 4 mm, the THz beam shows an elliptical shape which simply reflects an initial beam shape at the source point. As *L* increases, the magnitude profile becomes inhomogeneous particularly along the *x*-axis; a major peak becomes more narrowly defined, and satellite peaks are clearly discernible being located nearby. Interestingly, the beam magnitudes along the *y*-axis do not seem to undergo significant changes, sharply contrasting to the *x*-axis case.

To make a more direct comparison with the experimental results, we estimate the magnitude profiles of the THz beam as in the knife-measurements along the *x*- and *y*-directions. Figure 5(b–d) display the magnitude and its derivative profiles at three measurement positions, i.e., *L* = 4, 28, and 52 mm. At *L* = 4 mm, both quantities exhibit smooth position-dependent variations whereas the results along the *x*-axis reflect a broader profile as expected (Fig. 5(d)). As *L* increases, although the results along the *y*-axis remain with no discernible change, the profiles along the *x*-axis exhibit highly irregular position dependences; the derivative profiles become asymmetric and satellite peaks appear as expected from the amplitude distribution shown in Fig. 5(a). It should be noted that this simple simulation result catches most of the essential features of the experimental results.

Two-dimensional Gaussian profiles observed at *L* < 4 mm imply that the Gaussian magnitude distribution of the point dipoles plays an important role in determining the THz beam profile up to the certain distance in the far-field zone. To account for anisotropic non-Gaussian beam profiles observed at *L* > 4 mm, on the other hand, it is clear that we have to consider the phase distribution as well as the amplitude distribution of the point dipoles. Along the *x*-axis, several satellite peaks appear. They are attributed to the position-dependent phase retardation of the THz waves generated from each dipole due to the grazing incidence of the pumping beam. As soon as the surge-current-induced electric dipole is created, the THz wave is radiated as an electric dipole radiation with its oscillation phase given uniformly irrespective of the generation point (Fig. 1(a and b)). Since the grazing incidence leads to the difference in the travelling length from each generation point to the detection plane, it is natural to observe the interference effect particularly in the far-field region (See Fig. 1(a)). It should be noted that this situation contrasts to the grazing incidence reflection process; the point dipoles formed at the medium interface oscillate simply following the incident wave, and hence the phase relationship in the wavefront is readily recovered during the wave propagation after the reflection. This conclusion is supported by the observation of the Gaussian THz beam profile along the *y*-axis at the same value of *L*. Namely, along the *y*-axis, the phase of the point dipole or, equivalently, the THz wave is given homogeneously, and hence the THz beam profile is determined mainly by the magnitude distribution of the point dipoles following a propagation theory of the simple Gaussian beam (Fig. 1(b)). As we identify the interference effect in describing the evolution of THz beam profiles in the far-field regime, it is worthy to note the role of the initial size of the THz beam. Interestingly, we computationally observe that the evolution of the THz beam profile has a strong ${{\rm{\Phi }}}_{0}^{{\rm{THz}}}$-dependence; whereas the non-Gaussian beam profile is observed when ${{\rm{\Phi }}}_{0}^{{\rm{THz}}}$ = 1 mm, the Gaussian beam profile is maintained when ${{\rm{\Phi }}}_{0}^{{\rm{THz}}}$ is larger than 3 mm. Consistently with a common understanding of the interference effect for the wave, we consider that a diffraction-limited size of ${{\rm{\Phi }}}_{0}^{{\rm{THz}}}$ is another major factor leading to the non-Gaussian THz beam profile in the far-field regime.

## Conclusion

We examined how the beam profile of the surge-current-induced THz wave, generated at the surface of the unbiased GaAs, evolves as it propagates from the source point to the near-field zone, and also to the far-field zone. During its propagation, we observed a clear crossover behavior of the beam profile from the Gaussian shape in the near-field zone to the anisotropic non-Gaussian shape in the far-field zone. From the comparison with the simulation results based on the point-dipole radiation theory, we concluded that the THz wave properties are determined basically by the intensity profile of the pumping beam, incidence angle, and its size; the THz profile in the near-field zone is determined by the magnitude distribution of the point dipoles, i.e., the pumping beam intensity profile, and the THz profile in the far-field zone is influenced not only by the magnitude distribution but also by the phase distribution of point dipoles. As a result, we expect to observe a deviation from the Gaussian form more easily by increasing the incidence angle and also by reducing the pumping beam size particularly down to the diffraction-limit of the THz wave. We emphasize that this understanding can be applied to other experimental results characterizing beam profiles of THz waves. In the far-field zone, the THz wave has been reported to exhibit both the Gaussian and non-Gaussian shape^15,17,19,20^, and we consider that each result could be attributed to the phase contribution for the normal^15,17,19^ and grazing incidence^20^, respectively. In the near-field zone, on the other hand, the THz profile is determined rather by the pumping beam profile leading to the Gaussian distribution as observed^16^.
